# The role of probiotics in restoring and maintaining vaginal microbiome health: a review

**DOI:** 10.1128/iai.00011-26

**Published:** 2026-06-26

**Authors:** Binaya Krushna Sahu, Sujogya Kumar Panda, Utkalika Mallick, Smita Hasini Panda, Mahesh Chandra Sahu

**Affiliations:** 1 Centre for Biotechnology, Siksha ‘O’ Anusandhan (Deemed to be University) 177142, Bhubaneswar, Odisha, India; 2 Microbiology Division, ICMR-Regional Medical Research Centre 29727, Bhubaneswar, Odisha, India; 3 Department of Microbiology, Fakir Mohan University, Balasore, Odisha, India; University of Pittsburgh, Pittsburgh, Pennsylvania, USA

**Keywords:** *Lactobacillus*, *Bifidobacterium*, BV, VVC, UTIs, reproductive health, gestational diabetes, preterm labor

## Abstract

The vaginal microbiome is an important aspect of female reproductive health. The dominant microbial species of this ecosystem, *Lactobacillus*, offers protection from vaginal infections by maintaining lower pH levels and reducing potential pathogen colonization. Dysbiosis, or imbalance of the vaginal microbial ecosystem, has been associated with both common infections, such as bacterial vaginosis (BV), vulvovaginal candidiasis (VVC), and urinary tract infections (UTIs), and obstetric complications, including gestational diabetes, preterm labor, and obstetric anemia. This review provides an overview of the composition and function of the vaginal microbiota, emphasizing the role of *Lactobacillus* and other beneficial microbes and their mechanisms of action, including lactic acid and hydrogen peroxide production, competitive adherence, and immune modulation. Evidence from clinical trials supports the efficacy of these strains in reducing recurrence of BV, VVC, and UTIs. Additionally, emerging research shows promise for probiotic use in managing reproductive conditions such as gestational diabetes mellitus, preterm labor, and obstetric anemia. The review also discusses safety considerations, particularly in immunocompromised individuals, and the expanding interest in non-*Lactobacillus* genera like *Bifidobacterium* and *Bacillus*. Targeted probiotic interventions have great potential for restoring and maintaining a healthy vaginal microbiome, preventing recurrent infections, and helping improve reproductive outcomes. However, in order to incorporate these approaches in clinical practice, probiotic strains, delivery method, and dosage all need standardization.

## INTRODUCTION

The vaginal microbiota acts as a natural barrier against infections, which is essential to female health. It is predominantly composed of *Lactobacillus* species, which help as a first line of defense against infections by keeping the environment acidic and making it an unfriendly environment for many bacteria and fungi. These species achieve this pH balance by producing lactic acid, which inhibits harmful pathogens from growing (1–5). Dysbiosis can lead to the proliferation of harmful bacteria, increasing the risk of illnesses, such as bacterial vaginosis (BV), urinary tract infections (UTIs), and yeast infections (5, 6). The disturbed microbiome is related to a greater incidence of these diseases, which may further lead to problems, including increased risk of sexually transmitted infections (STIs), premature delivery in pregnant women, and chronic pelvic inflammatory disease (7).

**Fig 1:**
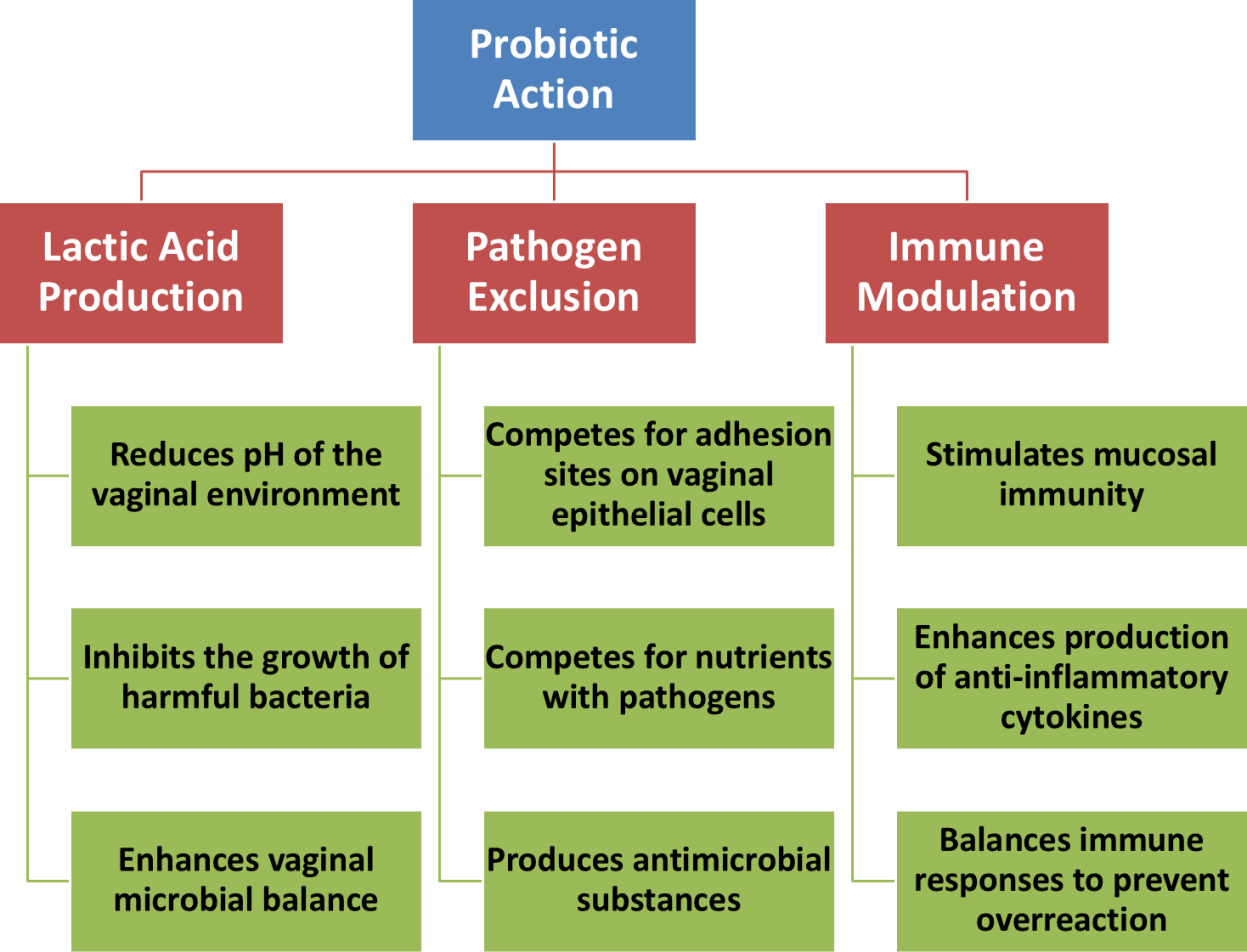
Probiotic action in promoting vaginal health.

Certain probiotics, often referred to as “friendly bacteria,” are live microorganisms that, when administered in adequate amounts through oral or vaginal routes, help maintain a balanced microbial environment (8). They are commonly found in fermented foods (yogurt, kefyr) and dietary supplements. The most effective probiotics for vaginal health include *Lactobacillus* strains such as *L. crispatus*, *L. rhamnosus*, *L. gasseri*, *L. jensenii*, *L. reuteri*, and *L. fermentum*, which have the abilities to colonize the vaginal epithelium and restore vaginal microbial balance (9). Probiotics support through several mechanisms (Fig. 1) that help maintain a balanced microbiome and protect against harmful pathogens (10). By attaching themselves to vaginal epithelial cells, they form a physical barrier, and accomplish competitive exclusion (11, 12). Additionally, they alter the immune system, boosting immunological responses and promoting the synthesis of immune-regulating compounds that inhibit the spread of pathogens (13). By encouraging the growth of *Lactobacillus*, a probiotic-rich diet can help maintain a healthy vaginal microbiota (9, 10). However, eating a lot of sugar can promote yeast overgrowth (14, 15). The pH and vaginal microbiota can be disturbed by certain hygiene habits, such as using harsh soaps or feminine hygiene products. Douching is associated with a higher risk of dysbiosis, as it changes the natural pH and removes beneficial bacteria (15).

Supporting the vaginal microbiota requires a more focused lifestyle advice like proper hydration, hygiene habits, and food habits. Preserving good *Lactobacillus* populations and maintaining an ideal pH can be achieved by avoiding douching, perfumed feminine products, and needless antibiotic usage. Consumption of probiotic-rich foods or supplements, particularly those containing *Lactobacillus* species, has been reported to support restoration of the vaginal microbiome by promoting re-establishment of protective, lactic acid-producing bacteria, especially following antibiotic use or hormonal disturbances (8–10). A robust vaginal microbiota is crucial for general female health and defense against infections.

## METHODOLOGY

This review was conducted using a narrative approach to summarize current knowledge on the role of probiotics in vaginal microbiome health. Relevant literature was identified through searches of electronic databases including PubMed, Scopus, and Google Scholar. Keywords such as “vaginal microbiome,” “probiotics,” “*Lactobacillus*,” “bacterial vaginosis,” “vulvovaginal candidiasis,” and “urinary tract infections” were used to retrieve relevant studies. Inclusion criteria comprised peer-reviewed original research articles, randomized controlled trials, clinical studies, and high-quality review papers focusing on vaginal microbiota composition, probiotic mechanisms, and clinical outcomes related to vaginal health. Studies published in English, with clear methodology and relevance to human health, particularly in reproductive-age women, were prioritized. Exclusion criteria included studies not directly related to the vaginal microbiome, those lacking experimental or clinical evidence, non-peer-reviewed articles, conference abstracts without full data, duplicate studies, and reports with insufficient methodological details or unclear outcomes. The selected studies were critically evaluated and synthesized to provide a comprehensive overview of probiotic roles, mechanisms, and clinical applications in maintaining and restoring vaginal microbiome balance.

## VAGINAL MICROBIOME: COMPOSITION AND INFLUENCING FACTORS

**Fig 2:**
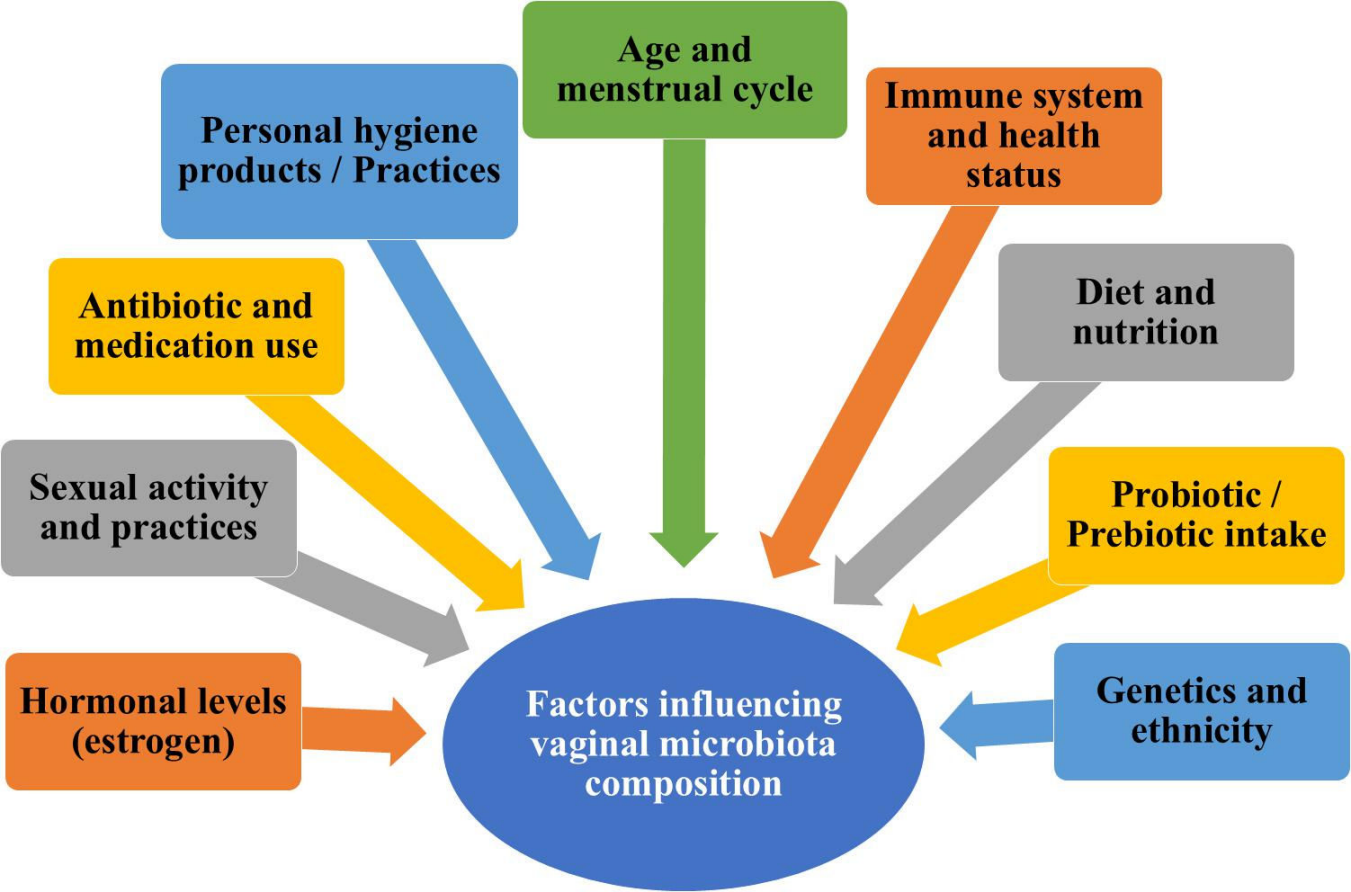
Factors influencing vaginal microbiota composition.

*Lactobacillus* species are the predominant microorganisms in the vaginal microbiome and play a crucial role in maintaining its health. By fermenting carbohydrates, *Lactobacillus* species, such as *L. crispatus, L. jensenii, L. gasseri*, and *L. iners* produce lactic acid, which inhibits pathogens by creating an acidic environment. Lactate dehydrogenase (LDH) uses NADH to convert pyruvate to lactic acid during fermentation, which lowers pH (16–18). This acidic condition suppresses the growth of pathogens, such as *Gardnerella vaginalis* and *E. coli*, which prefer neutral environments. The low pH also promotes the growth of beneficial bacteria and creates a balanced microbial ecosystem. This barrier helps prevent common infections like BV and UTIs (9). A healthy vaginal microbiome can vary from individual to individual and even alter within the same individual due to various factors (Fig. 2). Maintaining a *Lactobacillus-*dominated microbiome is mainly associated with lower rates of infection, better reproductive health and fewer complications during pregnancy.

**Fig 3:**
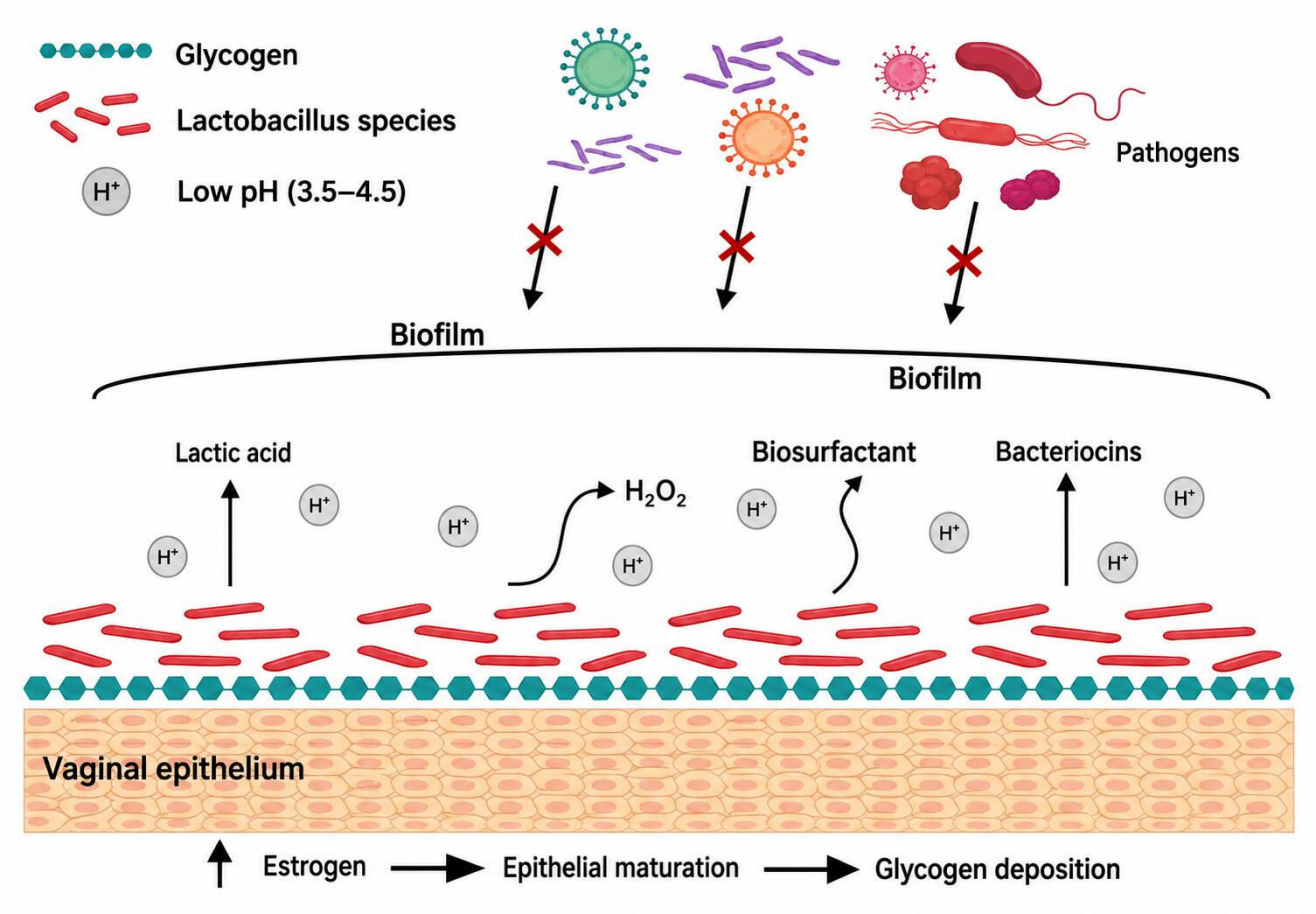
Protective function of estrogen-induced *Lactobacillus* activity in the vaginal microbiome stability. This figure shows how estrogen supports vaginal health by development of *Lactobacillus* species growth via epithelial maturation and glycogen deposits. Estrogen stimulates glycogen deposits in vaginal epithelial cells, which is utilized as a substrate for *Lactobacillus. Lactobacillus* bacteria ferment the glycogen-derived glucose into lactic acid, which is used to maintain a low vaginal pH (3.5-4.5) that inhibits pathogenic microbe growth. In addition to acidification, *Lactobacillus* species excrete antimicrobial substances, such as hydrogen peroxide (H_2_O_2_), biosurfactants, and bacteriocins, that prevent pathogenic biofilm formation and adhesion.

Various factors can affect the composition and stability of the vaginal microbiome, potentially leading to dysbiosis or microbial imbalance. Hormonal level changes, especially increased estrogen levels during the reproductive ages, support the growth of *Lactobacillus* since it increases glycogen in vaginal epithelial cells (6, 9). Estrogen also stimulates glycogen production for vaginal epithelial cells, where the *Lactobacillus* species may use it as a nutrient source. The action of enzymes converts glycogen to glucose, which *Lactobacillus* ferments to produce lactic acid without using oxygen, which helps maintain vaginal pH at 3.5-4.5, inhibiting the growth of potentially harmful bacteria or yeast (Fig. 3). In contrast, hormonal changes (those associated with menopause, menstruation, or pregnancy) can cause disturbances in the homeostasis of the vaginal microbiome (19). Antibiotics are important for the treatment of bacterial illnesses. Other factors that play an important role in microbial equilibrium include sexual activity, self-care hygiene practices, diet, immune status, use of antibiotics or medications, and the use of prebiotics or probiotics. Unique genetics and ethnicity may create variability in microbiota profiles, contributing to inter-individual differences (15, 20). However, they also disrupt the microbial community by indiscriminately killing the beneficial bacteria with the pathogens. This can include reducing the population of *Lactobacillus* and not having *Lactobacillus* present; thus, opportunistic infections of BV and yeast can occur.

## PROBIOTICS IN VAGINAL MICROBIOME HEALTH

*L. rhamnosus* GR-1 is a well-studied probiotic strain, especially for its beneficial effects on vaginal health. *L. rhamnosus* GR-1 adheres to vaginal epithelium, which supports colonization of healthy microbes. It produces lactic acid, which promotes an acidic vaginal pH, inhibiting the growth of potentially pathogenic microbes, such as *Gardnerella vaginalis*, a common pathogen associated with BV. This strain also has reported anti-inflammatory properties, which may reduce expressions of symptoms, such as irritation and discomfort, that may be associated with a vaginal infection (21–23). *L. reuteri* RC-14 is also a well-studied strain, frequently found alongside *L. rhamnosus* GR-1 in probiotic formulations, that is known for its positive benefits on vaginal health. It can survive within the acidic condition of the vagina and produces H_2_O_2_ to inhibit the growth of pathogenic microbes, such as *C. albicans*. When used simultaneously, *L. rhamnosus* GR-1 and *L. reuteri* RC-14 working together create an additive effect, with each one representing a unique individual in the vaginal microbiome to offer synergy to create balance and resilience (21–23). *L. crispatus* is a major vaginal species, and it produces lactic acid, which contributes to low pH and is associated with a reduced risk of BV and UTIs. It is found in women who have a lower susceptibility to sexually transmitted infections and is a marker of a normal vaginal microbiome. When lactobacilli are decreased and not naturally occurring, using *L. crispatus* can support the biotic balance process and promote vaginal health (24). *L. jensenii* can survive at a low pH in the vagina, and it produces lactic acid and antimicrobial substances that help maintain pH balance and help prevent infections. *L. jensenii* also produces biosurfactants that prevent pathogenic bacteria from adhering to the vaginal epithelium, acting as a natural barrier against pathogenic microorganisms from entering the vaginal epithelium (25). Like *L. crispatus*, *L. gasseri* is often found in women with fewer vaginal infections and is known to support a healthy, balanced microbiome. *L. gasseri* is well-known to have immune-enhancing effects, along with a number of other health benefits for both the vagina and gut. Its ability to persist in the human body makes it particularly favorable for long-term maintenance of vaginal health. *L. gasseri* is most commonly found in general health probiotic formulations, as well as formulations intended specifically for vaginal care, and researchers are picking up on increased benefit as *L. gasseri* paired with similar *Lactobacillus* strains always work together (5). The effects of probiotic strains that form benefits on vaginal ability (Table 1), along with other potential benefits like reducing BV and driving immune defense.

**TABLE 1:** Probiotic strains and their functions for vaginal health

Probiotic strain	Mechanisms of action	Reported benefits	References
*Lactobacillus rhamnosus* GR-1	Lactic acid production, adherence to vaginal cells	Reduces BV recurrence, anti-inflammatory	(21–23)
*Lactobacillus reuteri* RC-14	Hydrogen peroxide production, competitive exclusion	Inhibits *C. albicans* growth, enhances vaginal health	(21–23)
*Lactobacillus crispatus*	Lactic acid production, maintenance of low vaginal pH	Decreases risk of BV and UTIs	(9, 26)
*Lactobacillus jensenii*	Biosurfactant production, competitive adherence	Prevents pathogenic adhesion, supports immune modulation	(12, 19)
*Bifidobacterium bifidum*	Immune modulation, pH balance	Potential support for immune health and pathogen resistance	(10)

Non-*Lactobacillus* probiotics, such as *Bifidobacterium* species, are increasingly being explored for their potential role in supporting vaginal health, though their benefits are not yet as well-documented as those of *Lactobacillus*. *Bifidobacterium* strains help to sustain the balance of the microbiome by enhancing immune function and perhaps helping to improve resistance to pathogenic organisms. They play a role in shaping the vaginal microbiome by enhancing microbial diversity and producing short-chain fatty acids (SCFAs), such as acetate and propionate, which work to lower the vaginal pH and reduce the growth of harmful microbes (27, 28). There are promising effects on vaginal health beyond *Bifidobacterium* strains from other non-*Lactobacillus* genera, such as *Streptococcus*, *Enterococcus,* and *Bacillus. Streptococcus thermophilus* has been examined for its ability to adhere to vaginal epithelial cells and reduce harmful pathogen colonization. Species in the *Bacillus* genus are known for producing bioactive substances that enhance the stability of the vaginal microbiota by inhibiting the decline of detrimental microbes (2, 29). Recent work suggests that non-*Lactobacillus* strains may have a beneficial adjunctive role when *Lactobacillus* species alone are not appropriate or capable of restoring a healthy vaginal microbiome, and probiotics with non-*Lactobacillus* strains may have particular efficacy for recurrent BV or yeast infections because the addition of all non-*Lactobacillus* strains may offer broader and stronger protection against harmful pathogens (30). Research into non-*Lactobacillus* probiotics is still relatively nascent, but early findings demonstrate potential for more diverse microbial protection and overall vaginal health. Further studies on mechanisms of action, interactions with vaginal microbiota, and clinical effects will be necessary to clarify their role in providing support for vaginal health.

## CLINICAL EVIDENCE ON PROBIOTICS IN VAGINAL HEALTH

### Probiotics in the prevention and treatment of BV

**Fig 4:**
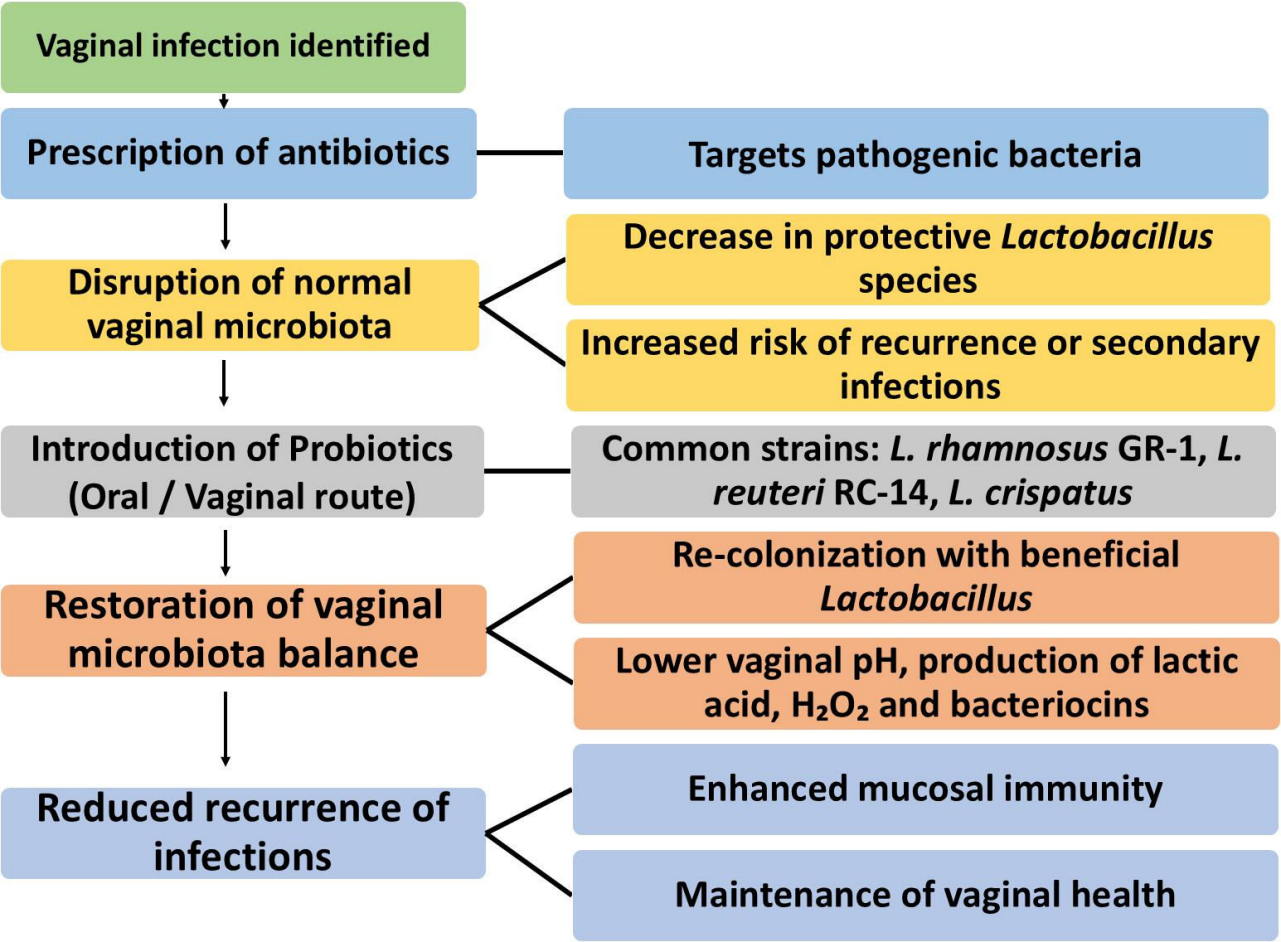
Stepwise approach of combined antibiotic and probiotic therapy for restoring vaginal microbiota and preventing recurrence of infections.

Bacterial vaginosis typically involves depletion of *Lactobacillus* species and proliferation of anaerobic bacteria, with *Gardnerella vaginalis* typically being the most notable (31). Prevalence of BV is substantial and as high as 39.5% in reproductive-age women in Ethiopia (32). Prevalence rates for BV tend to be higher in low- and middle-income countries and can reach over 50% in some areas (33). Probiotics have been effective for both treatment and prevention of BV by restoring the dominance of *Lactobacilli*. Even though antibiotics (metronidazole or clindamycin) are the first-line treatment for BV, probiotic supplementation can be used with antibiotics to facilitate recolonization of *Lactobacilli* and as a potential means of reducing the recurrence of BV (Fig. 4) (34, 35). Recent studies suggest that *L. crispatus* can be effective for treatment and prevention of BV; for example, Cohen et al. (26) demonstrated clinical effectiveness for *L. crispatus* where patients receiving *L. crispatus* intravaginally had 50% less chance of recurrence of BV in comparison to placebo treatments. Other studies have employed *L. rhamnosus* GR-1 and *L. reuteri* RC-14 orally and intra-vaginally and show a significantly lower recurrence of BV than controls, restored *Lactobacillus* dominance (23, 36, 37). These probiotics operate through their action on lactic acid production, competition for adhesion sites, and direct antimicrobial activity against pathogens associated with BV. Moreover, the use of probiotics in conjunction with antibiotics was shown to improve outcomes of antibiotic therapy. A meta-analysis from 2019 showed that women treated with antibiotics plus probiotics had a higher cure rate for BV than women treated with antibiotics only, thus supporting the role of probiotics in enhancing therapeutic efficacy and possibly preventing recurrence (38). Probiotics are increasingly used in the prevention and treatment of BV and restoration of vaginal microbiome health and possibly decreasing BV recurrences. More studies are required to determine the optimum formulation and delivery method for a probiotic.

### Yeast infections

On average, 75% of women around the world will have at least one occurrence of vulvovaginal candidiasis (VVC), most commonly caused by *C. albicans*, during their lifetime. Pregnancy, contraceptives, high-dose estrogen, diabetes, and immune suppression increase the rates of yeast infections (39). This yeast can flourish when a woman has a destabilized vaginal environment, especially without the presence of *Lactobacillus* (2). Probiotic strains, such as *L. rhamnosus* GR-1, *L. reuteri* RC-14, and *L. acidophilus* have been shown in research evidence to inhibit *Candida* and reduce symptoms of yeast infections. Specifically, studies show *Lactobacillus* strains inhibit *Candida* in several ways, one of which includes the production of lactic acid that lowers the pH levels, such as a lower pH that *Candida* cannot thrive. *Lactobacillus* species have also been shown to produce hydrogen peroxide and bacteriocins, which are known to be antifungal to *Candida* cells (40, 41). Women treated with probiotics during antifungal therapy for yeast infections had a significantly lower recurrence rate during a 6-month follow-up compared with women not treated with probiotics (42). Furthermore, the use of daily oral probiotic supplements was associated with decreased episodes of recurrent yeast infections, particularly in women with a history of frequent recurrentinfections (43). Probiotics increase *Lactobacillus* in the vaginal microbiome and enhance an environment better suited to prevent *Candida* overgrowth. Probiotics are a potentially novel non-pharmacological approach to treating VVC, maintaining the vaginal microbiome, and reducing yeast infections. They inhibit the growth of *Candida*, so they may be a helpful adjunct to traditional antifungal therapy; however, future studies should focus on dosing or probiotic formulation, as well as research related to the long-term use of probiotics in the management of VVC.

### Urinary tract infections (UTIs)

Recurrent urinary tract infections, most commonly involving uropathogens like *E. coli*, have been linked to alterations in a woman’s vaginal microbiota when they are at risk for recurrent infections (7). Probiotic strains, such as *L. rhamnosus* GR-1 and *L. reuteri* RC-14, have been shown to significantly reduce recurrent UTIs by restoring a normal *Lactobacillus* predominance and also inhibiting pathogenic bacteria from adhering to uroepithelial cells. These probiotics can colonize the urogenital tract, producing inhibitory byproducts lactic acid and hydrogen peroxide, which can impact uropathogen viability (44). A clinical trial published in 2017 found that women with recurrent UTIs, taking probiotic supplementation, experienced a significantly reduced recurrence compared with the placebo group (45). Moreover, probiotics have been shown to interact with antibiotics synergistically, possibly reducing antibiotic dosage and the development of antibiotic resistance (45). Probiotics also aid in immune regulation to assist the body in sensing and eliminating uropathogenic bacteria more effectively. There is evidence indicating *Lactobacillus* probiotics show promise as a strategy for preventing recurrent UTIs if used alongside standard care antibiotics (46). By promoting a healthy urogenital microbiome, hindering pathogen colonization, and enhancing host immunity, probiotics serve as a promising, sustainable adjunct in UTI prevention. Their complementary action with antibiotics may also reduce dependency on high-dose regimens and help mitigate resistance issues.

To summarize, standard treatments and probiotic support for BV, candidiasis, and UTIs reinforce the therapeutic integration of antibiotics/antifungals with microbiome-restoring probiotics to enhance clinical outcomes. Table 2 provides a clear and structured overview of the clinical applications of probiotics in the treatment of commonly encountered vaginal disorders such as BV, VVC, and UTIs. Specifically, clinical data available and collates the important outcomes from various clinical studies and trials, examining the successes of using different strains of probiotics, doses, and methods (oral vs vaginal) of delivery in real vaginal patients. For some of the research studies, especially those trials that utilized established and documented probiotic strains like *L. rhamnosus* GR-1 and *L. reuteri* RC-14, showed clear benefit, including decreased symptoms of the disorder, improved prevention of recurrent events, and restoration of healthy *Lactobacilli* in the vagina, reported longer follow-up times, and monitoring using modern technology to monitor the overall change in the vaginal microbiome, which enhances their validity.

**TABLE 2:** Clinical effects of probiotics in the treatment and prevention of BV, VVC, and UTIs

Condition	Probiotics used	Method	Results	Reference
BV	*Lactobacillus rhamnosus* GR-1 and *Lactobacillus reuteri* RC-14 (1 × 10⁹ CFU each)	125 premenopausal women with BV treated with metronidazole (500 mg BID, days 1–7), randomized to receive probiotics or placebo orally BID for 30 days; primary outcome measured at day 30 by Nugent score, symptoms, and sialidase test	Cure rate at day 30 was 88% in the antibiotic + probiotic group vs 40% in the antibiotic + placebo group (*P* < 0.001). The probiotic group had higher vaginal *Lactobacillus* recovery (96% vs 53%) and no BV recurrence by Nugent score.	(47)
BV	*Lactobacillus crispatus* CTV-05 (Lactin-V)	228 women aged 18–45 with recent BV diagnosis received vaginal metronidazole, then randomized (2:1) to receive either intravaginal Lactin-V or placebo for 11 weeks; primary outcome: recurrence by week 12; follow-up to week 24.	BV recurrence at week 12: 30% in Lactin-V group vs 45% in placebo (RR = 0.66, 95% CI: 0.441–0.87, *P* = 0.01). At week 24: RR = 0.73 (95% CI: 0.541–0.92). *L. crispatus* detected in 79% of treated women. Adverse events were similar in both groups.	(26)
BV	Vaginal tablet containing selected *Lactobacillus* strains	Double-blind, placebo-controlled clinical trial with 39 women diagnosed with BV; participants received either one vaginal tablet (probiotic or placebo) daily for 7 days; follow-ups were done immediately post-therapy and 2 weeks later.	Post-treatment: 100% of probiotic group free of BV (83% normal, 17% intermediate flora) vs 12% in placebo (*P* < 0.001). At 2 weeks: 61% success in treatment group vs 19% in placebo (*P* < 0.05). Significant symptom reduction, especially in vaginal odor.	(48)
BV	Vaginal capsules containing *Lactobacillus rhamnosus*, *L. acidophilus*, and *Streptococcus thermophilus*	Randomized, placebo-controlled trial; 120 Chinese women with recurrent BV received either probiotic or placebo vaginal capsules for 3 weeks (7 days on, 7 days off, 7 days on); followed up for 11 months	BV recurrence significantly lower in the probiotic group (15.8%) vs placebo (45.0%, *P* < 0.001); *G. vaginalis* incidence also significantly lower (3.5% vs 18.3%, *P* = 0.02); benefits sustained through 11 months; no adverse events reported	(49)
BV	Oral intake of *Lactobacillus rhamnosus* GR-1 and *Lactobacillus fermentum* RC-14	Randomized, placebo-controlled trial on 59 healthy women with BV. Daily oral probiotic supplementation was given for 2 months. Diagnosis based on Nugent score and PCR-DGGE.	Restoration of lactobacilli-dominant microbiota is observed only in the probiotic group, not in controls; BV remains common in healthy postmenopausal women and involves diverse pathogens beyond *Gardnerella*. The FemExam pH system was unreliable.	(50)
BV	Oral capsules containing *Lactobacillus rhamnosus* GR-1 and *Lactobacillus reuteri* RC-14	Randomized, double-blind, multicentric, placebo-controlled trial involving 544 women diagnosed with BV. Participants received either probiotics or a placebo daily for 6 weeks, with evaluations at weeks 6 and 12.	After 6 weeks, 61.5% in the probiotic group vs. 26.9% in the placebo group had restored microbiota (*P* < 0.001). At 12 weeks, 51.1% in the probiotic group retained normal microbiota vs 20.8% in the placebo group (*P* < 0.001).	(37)
VVC	*Lactobacillus acidophilus* LA-5, 1 × 10⁹ CFU/g, 30 oral capsules	Triple-blinded RCT, 80 married women aged 18–49 with clinically and microbiologically confirmed VVC; Group 1: Single dose of fluconazole (150 mg) + probiotic placebo; Group 2: Probiotic capsules for 30 days + fluconazole placebo. Follow-up at ~30 and ~60 days post-treatment.	No significant difference in negative culture rate at day 30 (*P* = 0.127). At day 60, the fluconazole group had a significantly higher negative culture rate (*P* = 0.016). Fluconazole was more effective in reducing abnormal discharge, erythema, and pruritus at follow-up. No difference in burning, dysuria, or dyspareunia. Probiotics had comparable short-term symptom relief but were less effective in preventing recurrence.	(51)
VVC	*Lactobacillus rhamnosus* GR-1 and *Lactobacillus reuteri* RC-14 (two capsules daily for 4 weeks)	Randomized, double-blind, placebo-controlled trial with 55 women diagnosed with VVC; all treated with a single 150 mg fluconazole dose plus either probiotic or placebo capsules for 4 weeks.	The probiotic group showed significantly less vaginal discharge (10.3% vs. 34.6%, *P* = 0.03) and lower yeast presence by culture (10.3% vs. 38.5%, *P* = 0.014) at 4 weeks compared with the placebo. Probiotics enhanced antifungal treatment efficacy.	(36)
VVC	Vaginal and oral probiotics (unspecified strains) plus fluconazole	Double-blind, randomized, placebo-controlled trial; 76 women with VVC; 4 weeks treatment	Significant symptom reduction in probiotic group; higher mycological cure (68.4% vs 46.9%, *P* = 0.184); significant culture reduction after adjusting for fluconazole resistance	(52)
VVC	Vaginal capsules with *Lactobacillus gasseri* LN40, *L. fermentum* LN99, *L. casei* subsp. *rhamnosus* LN113, *Pediococcus acidilactici* LN23	Randomized double-blind placebo-controlled trial; 95 women post-conventional treatment; vaginal capsules administered 5 days; follow-up after 2–3 days, 1st and 2nd menstruations, and 6 months	89% colonization of LN strains 2–3 days after administration (0% placebo); colonization dropped to 53% after one menstruation; 9% still colonized at 6 months; intervention group showed less malodorous discharge and somewhat fewer recurrences (no significant difference in cure rates)	(53)
VVC	SynForU-HerCare (lactobacilli probiotic; two capsules/day, 9.5 log CFU/capsule)	Randomized, double-blind, placebo-controlled trial; 78 pregnant women with VC; probiotic or placebo administered for 8 weeks; vaginal and fecal microbiota profiled at weeks 0, 4, and 8	Probiotic prevented significant microbial community shifts in high vaginal region seen in placebo group (decrease in *Lactobacillus* and increase in *Prevotella* and *Atopobium*). Probiotics also prevented reduction in gut Firmicutes abundance and related taxonomic levels, maintaining healthier vaginal and gut microbiota during VC.	(54)
UTI	*Lactobacillus GG* (Dicoflor, 6 × 10⁹ CFU once daily)	Double-blind randomized controlled trial in 12 NICUs, 585 preterm infants (<33 weeks gestation or <1,500 g birthweight), probiotic or placebo administered from first feed until discharge	No significant reduction in incidence of UTIs (3.4% vs 5.8%), NEC (1.4% vs 2.7%), or bacterial sepsis (4.4% vs 3.8%) in probiotic group compared with placebo. Seven days of supplementation was not effective in reducing these infections in preterm infants.	(55)
UTI	*Lactobacillus acidophilus* and *Bifidobacterium lactis* (10^7^/mL, 0.25 ml/kg three times daily) + Nitrofurantoin (1 mg/kg daily)	Randomized clinical trial with 85 children assigned to probiotic +antibiotics (group I) or antibiotics alone (group II). Monthly urine exams for UTI incidence over 3 years.	Incidence of febrile UTIs was significantly lower in probiotic + antibiotics group in the last year (0.00 ± 0.00 vs 0.13 ± 0.40; *P* = 0.03). No significant differences in overall RUTI incidence or afebrile UTI incidence between groups.	(56)
UTI	Probiotic FamiLact capsules (500 mg daily) plus antibiotics vs antibiotics alone	Randomized clinical trial with 162 women diagnosed with UTI, randomized 1:1 to probiotic + antibiotics or antibiotics alone for 6 months. Recurrence tracked at 3 and 6 months.	At 3 months: Recurrence was 5.0% in probiotic group vs 53.1% in antibiotic-only group (*P* < 0.001). At 6 months: 2.5% vs 22.5% (*P* < 0.001). No significant difference in recurrence at 6-month follow-up (*P* = 0.37).	(45)
UTI	*Lactobacillus* standard strains (*L. casei*)	*In vitro* microplate assay to test antagonistic effects of three *Lactobacillus* strains against UTI isolates from 600 samples with ≥10,000 cfu/mL. Multidrug resistance tested, plasmid curing performed to identify resistance gene origin.	No antagonistic effect against *Enterococcus*, *Enterobacter*, *K. pneumoniae* (all highly antibiotic resistant). Inhibitory effect observed only against multidrug-resistant *E. coli*. *L. casei* was most effective. Plasmid curing showed ampicillin resistance likely plasmid-mediated and not inhibited by probiotic treatment.	(57)
UTI	Oral lactic acid bacteria + Bifidobacteria; Vaginal Lactobacilli	Double-blind, placebo-controlled RCT with 174 premenopausal women randomized into four groups (placebo, oral probiotic, vaginal probiotic, both) over 4 months; followed up for 1 year	Vaginal (G3) and combination (G4) groups had significantly fewer symptomatic UTIs (1.06 and 1.07 vs 2.1 in placebo); extended time to first UTI (123.8 and 141.8 days vs 69.3); well tolerated	(58)
VVC	*Lactobacillus acidophilus* GLA-14, *Lactobacillus rhamnosus* HN001, and bovine lactoferrin	Randomized controlled trial with 48 women diagnosed with VVC and history of recurrence. All participants received clotrimazole and were randomized to receive probiotic +l actoferrin or placebo for 15 days (induction phase), followed by 6-month maintenance (10 days/month).	Probiotic + lactoferrin group showed significant reduction in symptoms (itching and discharge) at 3 and 6 months. Recurrence rates were significantly lower: 33.3% vs 91.7% at 3 months; 29.2% vs 100% at 6 months.	(43)
VVI	*Bifidobacterium bifidum*, *B. lactis*, *Lactobacillus acidophilus*, *L. paracasei*, *L. rhamnosus*, *Streptococcus thermophilus*	Randomized, double-blind, placebo-controlled trial; 47 pregnant women post-treatment for VVIs received either two probiotic capsules/day or placebo until delivery; vaginal microbiota and recurrence tracked monthly and after complaints	No significant difference in VVI recurrence between probiotic and placebo groups; probiotic strains did not colonize the vagina; no effect on pregnancy outcomes	(59)
BV	Selected oral *Lactobacillus* strains	Randomized, double-blind, placebo-controlled trial with 644 asymptomatic pregnant women (<20 weeks gestation, Nugent score >3, pH ≥4.5). Probiotic or placebo given until 24–26 weeks gestation. Primary outcome: spontaneous preterm delivery (<34 or <32 weeks); secondary: neonatal morbidity.	No statistically significant difference in preterm birth at <34 or <37 weeks (RR: 0.33 and 0.49, respectively; *P* > 0.05). Minor neonatal complications were rare. Study underpowered to confirm efficacy.	(60)
BV	*Lactobacillus crispatus* CTV-05 (LACTIN-V)	24 women with BV treated with metronidazole gel, then randomized 3:1 to LACTIN-V or placebo for five consecutive days + 2 weekly doses. 16S rRNA qPCR tracked 9 BV-associated bacteria and *L. crispatus* colonization.	61% colonized with CTV-05 by day 28. Higher levels of *G. vaginalis* and *Atopobium spp*. were associated with failure to colonize. Endogenous *L. crispatus* at baseline and sexual activity during the study significantly impaired LACTIN-V colonization (*P* = 0.003 and *P* = 0.018, respectively).	(61)
BV	10-day course of intravaginal probiotic capsules (strain not specified)	Multicenter RCT; 358 women received probiotics, 358 received 7-day oral metronidazole. Follow-ups at 1st, 2nd, and 4th week for cure rate, and at 2nd, 3rd, and 4th month for recurrence in those cured.	No significant difference in cure rates (week 4 OR 1.338, *P* = 0.071). However, probiotics significantly reduced recurrence rates (OR 0.212-0.119, *P* = 0.000). Adverse events were minimal and comparable.	(62)
BV	*Ecologic Femi*+ vaginal capsule (multi-species Lactobacillus +one Bifidobacterium); *Gynophilus LP* (L. rhamnosus 35)	RCT (*n* = 68; 4 groups of 17); HIV-negative, non-pregnant Rwandan women post-metronidazole; randomized to: counseling only (control), or counseling +oral metronidazole, Ecologic Femi+, or Gynophilus LP for 2 months. Vaginal microbiota assessed by Nugent score, qPCR, and sequencing.	BV incidence per person-year: Control = 10.18, Metronidazole = 1.41 (*P* = 0.004), Ecologic Femi+ = 3.58 (*P* = 0.043), Gynophilus LP = 5.36 (*P* = 0.220). Both metronidazole and Ecologic Femi+ increased *Lactobacillus* dominance and reduced BV anaerobes. Effects faded after stopping intervention.	(63)

## CLINICAL ROLE OF PROBIOTICS IN REPRODUCTIVE AGE WOMEN

### Gestational diabetes mellitus (GDM)

Gestational diabetes mellitus (GDM) affects approximately 1%–20% of pregnancies worldwide and is associated with adverse maternal and fetal outcomes, including increased risks of macrosomia and cesarean delivery (64). Probiotics are thought to benefit GDM management by improving insulin sensitivity, reducing inflammation, and modulating the gut microbiota (9). Lindsay et al. (65) found that supplementation with *Lactobacillus salivarius* significantly reduced fasting blood glucose levels and insulin resistance in women with GDM. Another study by Karamali et al. (66) investigated the effects of probiotic supplementation on pregnant women with GDM. Sixty primigravida women aged 18–40 years were assigned to receive either probiotic capsules (containing *Lactobacillus acidophilus*, *L. casei,* and *Bifidobacterium bifidum*) or placebo for 6 weeks. The probiotic group showed significant improvements in glycemic control (lower fasting glucose, insulin levels, and insulin resistance) and lipid profile (reduced triglycerides and VLDL cholesterol). However, other lipid parameters remained unchanged. Further large-scale studies are needed to standardize strains and dosages for clinical use in GDM management.

### Preterm labor

Preterm labor, defined as labor before 37 weeks of gestation, complicates 12.5% of all deliveries in the USA and is the leading cause of perinatal mortality and morbidity, responsible for up to 75% of perinatal deaths (67, 68). Infection and inflammation are significant risk factors for preterm labor, and recent research has examined whether probiotics can help reduce these risks by influencing the maternal immune system and vaginal microbiota (9). Prodan-Barbulescu et al. (69) reported that an imbalance in vaginal microbiota was associated with an increased risk of preterm birth that originated with altered *Lactobacillus*-dominant microbiota. Another study by Yang et al. (70) demonstrated that the cell-free supernatant of the probiotic *Lactobacillus rhamnosus* GR-1 significantly attenuated lipopolysaccharide-induced inflammation and reduced the incidence of preterm birth in pregnant mice. The probiotic supernatant decreased the production of several pro-inflammatory cytokines and chemokines in maternal and intrauterine tissues, suggesting its potential therapeutic role in preventing infection-associated preterm labor through modulation of systemic and local inflammatory responses.

### Obstetric anemia

Obstetric anemia, commonly due to iron deficiency, creates risks to both maternal and fetal health (71). Anemia in pregnancy affects about 40% globally, with a prevalence of about 50%-60% in low- and middle-income countries due to poor nutrition, infectious diseases, and lack of health service access. High-income countries report a much lower burden of anemia in pregnancy, with prevalence around 10%-20%. South Asia and sub-Saharan Africa are regions of high prevalence, which can partly be linked to malnutrition (iron) and infectious diseases (72). Recent investigations into the use of probiotics in conjunction with iron supplementation have found that probiotics not only act to promote gut health but also improve microbiota composition, which may be an important factor in improving iron absorption. Hoppe et al. (73) conducted a study using *L. plantarum* on pregnant women, leading to significant increases in iron absorption. Koker et al. (74) then explain that in previous research, when *Lactobacillus* strains were combined with probiotics, participants achieved markedly improved iron absorption and a lower risk of developing anemia. Probiotics could be a promising countertreatment against obstetric anemia since dysbiosis of the gut has been shown to disrupt iron metabolism.

Based on the available evidence, probiotics are likely to be helpful in the management of preterm labor, GDM, and obstetric anemia. The studies emphasize the therapeutic potential of certain probiotic strains, but there is a need for further studies to determine whether they are effective in managing these conditions, how to optimally dose probiotic strains, and their potential limitations. Ultimately, to summarize clinical studies (Table 3) of the use of probiotics for managing gestational diabetes mellitus, preterm labor, and obstetric anemia in recent years.

**TABLE 3:** Clinical studies on the use of probiotics in managing gestational diabetes mellitus, preterm labor, and obstetric anemia

Condition	Probiotic strains	Method	Results	Reference
Gestational diabetes mellitus	Probiotic yogurt containing *Lactobacillus* strains	84 GDM women in a double-blind, placebo-controlled trial; 300 g/day probiotic or ordinary yogurt for 8 weeks	Significant reductions in fasting and postprandial blood glucose and HbA1c in the probiotic group; fewer macrosomic births	(75)
Gestational diabetes mellitus	*Lactobacillus rhamnosus* HN001 (6 × 10⁹ CFU/day)	Double-blind, randomized, placebo-controlled parallel trial conducted in New Zealand (Wellington and Auckland) in 423 pregnant women with a personal or partner history of atopic disease. Intervention began at 14–16 weeks gestation. GDM was assessed at 24–30 weeks using IADPSG and NZ definitions.	HN001 group had a lower GDM prevalence using the NZ definition (2.1% vs 6.5%, *P* = 0.03). A trend toward lower GDM rates was observed using the IADPSG definition (RR = 0.59, 95% CI 0.32–1.08, *P* = 0.08). Significant reduction in GDM risk was observed in women aged ≥ 35 years (RR = 0.31, *P* = 0.009) and those with a history of GDM (RR = 0.00, *P* = 0.004). HN001 may be especially effective in high-risk groups.	(76)
Preterm labor	Vaginal probiotics containing four *Lactobacillus* strains	Randomized, prospective, longitudinal, double-blind, placebo-controlled multicentric trial (Oct 2017–Aug 2022) across seven centers in Spain; 200 pregnant women at risk of preterm birth randomly assigned to receive probiotics or placebo. Primary outcome: preterm birth before 37 weeks.	No statistically significant differences between probiotic and placebo groups in terms of spontaneous preterm birth, gestational age at delivery, neonatal complications, or vaginal microbiota composition. No serious adverse events reported.	(77)
Preterm labor	*Lactobacillus rhamnosus* GR-1 supernatant (GR-1 SN)	*In vivo* mouse model (pregnant CD-1 mice); intrauterine injection of lipopolysaccharide (LPS) with or without prior treatment with GR-1 SN. Measured PTB rates, cytokine/chemokine levels in maternal plasma, myometrium, placenta, amniotic fluid, and plasma progesterone. Statistical significance via ANOVA.	GR-1 SN reduced LPS-induced PTB by 43%. Significantly lowered inflammatory cytokines (IL-1β, IL-6, TNF-α, IL-12) and chemokines (CCL4, CCL5) in maternal and fetal tissues. Progesterone levels were reduced after LPS injection regardless of GR-1 SN treatment. No change in fetal sex ratio. GR-1 SN showed promise in attenuating inflammation-related PTB.	(70)
Obstetric anemia	*Lactiplantibacillus plantarum* 299v (Lp299v) (10¹° CFU) with 4.2 mg iron, 12 mg ascorbic acid, and 30 µg folic acid	Randomized, double-blind, placebo-controlled trial in 326 non-anemic pregnant Swedish women. Participants received Lp or placebo twice daily from gestational week 10–12 until end of pregnancy or iron therapy. Primary endpoint: serum ferritin at week 28. Other parameters: iron deficiency, anemia, transferrin receptor, total body iron.	Lp supplementation significantly attenuated ferritin decline at weeks 28 and 35. Reduced iron deficiency (59% vs 78%) and iron deficiency anemia (7.4% vs 21%) at week 35. Improved soluble transferrin receptor levels and total body iron. No adverse effects on gestational length or birthweight. Comparable safety profile to placebo.	(78)

## SAFETY AND CONSIDERATIONS

### Potential side effects

Probiotics are widely considered safe and are typically well-tolerated. However, minor gastrointestinal symptoms, such as bloating, gas, and mild abdominal discomfort, are sometimes reported in the initial phase of use, as the body adjusts. These symptoms generally resolve within a few days of consistent use. For individuals sensitive to ingredients in probiotic formulations, such as dairy, mild allergic reactions like itching, rash, or respiratory discomfort may occur. In such instances, discontinuing the probiotic and consulting a healthcare provider is advised (79).

### Considerations for immunocompromised individuals

For individuals with weakened immune systems, such as those with autoimmune diseases, undergoing chemotherapy, or on immunosuppressive medications, probiotics should be used with caution. Probiotics provide numerous health benefits, but reports have described rare serious infections, namely *L. bacteremia*, in patients who are immunocompromised (80). Stavropoulou and Bezirtzoglou (81) stated the infection risk is very low, but is still significant enough to be considered; therefore, it is important for healthcare professionals to evaluate the risk versus harms in each situation, where consideration may be taken to provide a lower dose or avoid entirely in significant immunocompromised patients.

### Strain-specific risks

Recent studies indicate that certain probiotic strains may introduce additional risks for immunocompromised patients. For example, *L. rhamnosus* GG is commonly used commercially, and following the occasional reports of bacteremia associated with it, the majority of cases are seen in patients with pre-existing risks (82). In a case of infection reported by Kothari et al. (83), the authors noted that the infection was with *L. rhamnosus* GG. This is a reminder that selecting certain strains is important when considering the use of probiotics in patients with immunocompromising conditions. *S. boulardii* has also been associated with fungemia, also in patients with central venous catheters (84). When working with patients at high risk for infections, this further emphasizes the importance of best selecting strains based on historical safety data.

In such groups, administration of probiotics should occur under medical supervision and with the careful selection of strains with limited evidence of safety. Although the efficacy of probiotics among high-risk individuals is uncertain, carefully selected short-term control may be acceptable when the potential benefits exceed risk. However, more evidence is needed to develop strain-specific standards for probiotic use to inform clinical use in immunocompromised patients.

## FUTURE DIRECTIONS ON NON-*LACTOBACILLUS* PROBIOTICS

While *Lactobacillus* strains are prominent in research in relation to vaginal health, we have opportunities to expand to other non-*Lactobacillus* probiotics to improve therapeutic efficacy and provide treatment alternatives. For more investigations, we recommend evaluating the contributions of other beneficial genera (*Bifidobacterium*, *Streptococcus,* and *Bacillus*) to understand how their actions can help rebalance the vaginal microbiota. Data show us that, for example, *Bifidobacterium* strains can function synergistically with *Lactobacillus* with an additional route to restore the vaginal ecosystem and enhance protection against infections (85). By studying other probiotics, we can provide more specific targeted interventions where patients are unresponsive to or unable to tolerate conventional *Lactobacillus*-based vaginal therapies. Furthermore, we can better understand how other non-*Lactobacillus* strains interact with the vaginal microbiome, and this potentially contributes to future personalized therapies that consider individual microbial composition. Improvements in delivery methods, such as intravaginal suppositories or controlled-release capsules should strengthen the stability and favorable therapeutic characteristics of alternative strains. These probiotics may be especially useful for unique populations, such as immunocompromised patients, where select strains may have a decreased risk of infection while enhancing microbiome resiliency. Ongoing research into these less-explored strains has the potential to broaden the scope and precision of probiotic therapies for vaginal health.

## CONCLUSION

Probiotics are an exciting option for restoring and maintaining the vaginal microbiome and for the prevention and management of common conditions like bacterial vaginosis, vulvovaginal candidiasis, and urinary tract infections. Selected *Lactobacillus* strains consistently have shown efficacy, including inhibition of pathogen growth, improvements in host immunity, and improving treatment when co-administered with antibiotics. While there is current evidence for the benefit of probiotics, further clinical trials are needed to develop standardized guidelines in terms of selected non-*Lactobacillus* strains, quantity, and duration of administration, so non-*Lactobacillus* probiotics can be fully exploited for clinical benefits.

## Data Availability

All data supporting the findings of this study are included within the manuscript.
